# Predictability of lower incisor tip using clear aligner therapy

**DOI:** 10.1186/s40510-022-00433-4

**Published:** 2022-11-07

**Authors:** Julia Meri Smith, Tony Weir, Austin Kaang, Mauro Farella

**Affiliations:** 1 Discipline of Orthodontics, University of Otago, PO Box 56, Dunedin, New Zealand; 2 Discipline of Orthodontics, University of Queensland, Brisbane, Australia; 3 Department of Surgical Sciences, University of Cagliari, Cagliari, Italy

**Keywords:** Lower incisor, Tip, Invisalign^®^, Clear aligners, Clear aligner therapy, Orthodontic tooth movement, Root movement, Attachments

## Abstract

**Background:**

Uprighting incisors is particularly important with clear aligner therapy as incisor tip determines the mesio-distal space needed in the arch, and consequently the fit of the aligner. The objective of this study was to investigate the accuracy of ClinCheck^®^ software to predict lower incisor tip by comparing digitally prescribed movements with actual clinical outcomes and to determine whether the presence of a vertically orientated rectangular composite attachment influences the efficacy of incisor tip.

**Methodology:**

This retrospective study included 66 lower incisors from 42 non-extraction adult patients treated using the Invisalign^®^ appliance. Twenty-one incisors had vertical attachments, while 45 incisors did not have any attachments. Lower incisor tip was measured at T0 (pre-treatment), T1 (predicted post-treatment) and T2 (achieved post-treatment) on digital models using metrology software. The change in position from T0 to T1 and T0 to T2 was measured from the estimated centre of resistance (C_Res_) of each tooth. The estimated centre of rotation was plotted relative to the C_Res_ to describe the type of orthodontic tooth movement (OTM) predicted and achieved.

**Results:**

Predicted incisor tip and achieved incisor tip were positively correlated (*R*^2^ = 0.55; *p* < 0.001). For every degree of tip planned 0.4 degrees of tip was achieved. The presence of an attachment resulted in 1.2 degrees greater tip (*F* = 3.7; *p* = 0.062) and 0.5 mm greater movement of the predicted apex of the tooth (*F* = 4.3; *p* = 0.042) compared with the no attachment group. The type of OTM achieved differed from the type predicted. Sixty-seven percent of incisors investigated were predicted to move by root movement, while 46% achieved this type of movement.

**Conclusions:**

The amount of lower incisor tip achieved was on average substantially less than the ClinCheck^®^ displayed. Vertically orientated rectangular attachments are recommended where large root movement is planned, and their presence slightly improves apex movement.

## Background

The therapeutic position of lower incisors in the sagittal plane remains a controversial subject in orthodontics [1]. In contrast, the position of the lower incisors in the coronal plane, as represented by their correct angulation or mesio-distal tip, has scarcely been investigated [2–4]. Andrews’ seminal paper describes the optimum mesio-distal tip as the “second key of occlusion”, as this determines the amount of mesio-distal space (arch length) required for alignment [4]. It also contributes to anterior aesthetics and, ultimately, posterior occlusion [2, 4, 5].

The achievement of a correct mesio-distal tip has become even more relevant with the increasing popularity of clear aligner treatment (CAT). Not only does the tip influence the final occlusion, but it also has a considerable effect on the fit of the aligner. If one or more teeth fail to upright and subsequently require more space than planned, the aligner will become distorted. Initially, this distortion, commonly referred to as a “tracking” issue, may not be detected. However, as these errors accrue throughout treatment, the adaption of the polymer to the teeth becomes problematic, and the biomechanics of the aligner may be compromised. Unwanted or inefficient tooth movements resulting in unsatisfactory treatment outcomes may then result.

Despite the seemingly rapid uptake of CAT since the inception of Invisalign^®^ (Align Technology, Santa Clara, California, USA) in the late 1990s, limitations with regard to biomechanics and reliability of orthodontic tooth movement (OTM) remain. It is well documented that the proposed tooth movements shown in the digital planning software do not express in their entirety [6–13]. A recent follow-up prospective clinical trial found a mean accuracy of movements predicted by proprietary software to be 50% [14]; this represents only a modest improvement from the 41% accuracy reported ten years prior [8].

One of the fundamental biomechanical challenges of CAT is controlling root movements [15, 16]. Previous studies have shown that root movements with CAT are under-expressed compared to crown movement [17–21]. Despite advances in CAT, it remains difficult to routinely achieve adequate root movement without the use of auxiliaries or extensive and time consuming refinements.

Root tip occurs when a high moment-to-force ratio is applied to the tooth, and its centre of rotation (C_Rot_) is located approximately at the incisal edge [22]. It requires two equal and opposite forces (*i.e.* force couple) acting on the crown along different lines of action. The result of this couple is that the forces cancel each other out, leaving a “pure” moment acting, which rotates the tooth [23]. Rectangular vertical attachments are often recommended to increase the surface area available for an effective couple with a greater moment arm [24]. There is some preliminary evidence to suggest vertical attachments may increase bodily distalisation of maxillary molars [25]. However, most attachment recommendations are based on anecdotal clinical experience rather than evidence. In theory, attachments should facilitate mesio-distal root uprighting, as well as bodily movement in the mesial or distal direction [26], but so far this principle has not been validated by independent clinical data. There is very little research available to verify that tooth root movements are being achieved [27], and there is a lack of evidence supporting the recommendation of each attachment.

To the best of our knowledge, the effect of vertical rectangular attachments on mandibular incisor tip efficacy with the Invisalign^®^ appliance has not been investigated. A better understanding of factors influencing the effectiveness of incisor tip with clear aligners is critical to improving occlusal outcomes, aligner fit, and the overall quality of treatment results.

This study aims to investigate the accuracy of Invisalign^®^ software predictions compared to the actual clinical outcome for lower incisor root tip and to determine whether the presence of vertical attachments improves the efficacy of incisor tip.

## Methods

This retrospective study was approved by the University of Otago Ethics Committee (HD20/004). The patients included in this study were treated between 2013 and 2019. Data were collected and analysed over a two-year period (2020–2021) in a university setting. The research report conforms to STROBE guidelines for observational studies.

### Patient sample

The patient sample was selected from an independent database of Invisalign^®^ cases compiled by a network of specialist orthodontists. At the time of the data collection, the database consisted of approximately 7000 Invisalign^®^ cases treated by private practising orthodontists in Australia and New Zealand, each with at least ten years of experience in CAT. Patients treated with the Invisalign^®^ appliance were selected according to the following inclusion criteria: adult age (*i.e.* > 18 years); Class I occlusion with minimal anterior–posterior movement planned; mild crowding; a minimum set of 14 aligners; aligners made with SmartTrack^®^ material; and at least one mandibular incisor with root tip planned equal to or greater than five degrees as displayed in the ClinCheck^®^ Tooth Movements Table (TMT). The exclusion criteria were: tooth extractions planned as part of orthodontic treatment; extensive tooth crown restorations made during treatment; interproximal reduction (IPR) of lower incisors > 0.2 mm per contact; absence of final intraoral scans; attachments remaining on the lower incisor in the final intraoral scan; and use of intermaxillary elastics or hybrid appliances (*e.g.* auxiliaries, power arms, brackets).

The sample size was estimated based on previously published data of standard deviation for incisor root movements (SD 4.9 degrees) [28]. By setting type I error at 0.05 and type II error at 0.10 (*i.e.* 90% power), it was estimated that 44 cases were sufficient to detect a clinically relevant difference of root movement equal to or greater than five degrees. The entire database was screened for the inclusion and exclusion criteria by a single examiner (TW). Sixty cases were included for screening by a second examiner (JS). Of those 60 cases, a further 18 cases were excluded due to: excessive IPR, use of intermaxillary elastics, attachments being present on the lower incisors in the final intraoral scan and corrupt ClinCheck^®^ files.

The final sample consisted of 42 patients (28 female, 14 male), from which 66 incisors were identified as eligible and included in the study. The descriptive statistics of the sample is presented in Table 1. The vast majority of patients changed their aligners every two weeks (71%), and the mean number of aligners was 27.6 (SD 9.4). Twenty-one incisors (32%) had vertical rectangular attachments, while 45 incisors (68%) had no attachment present. The configuration of vertical attachments varied from 3 to 5 mm (Table 1).

**Table 1:** Descriptive statistics for the sample investigated

	*N*	%
*Patients*		
Female	28	67
Male	14	33
Mean age (range)	34y9m (18y-77y7m)	
*Lower incisor*		
32	22	33.3
31	14	21.2
41	10	15.1
42	20	30.3
*Wear time prescription*		
1 week	12	29
2 weeks	30	71
*Vertical rectangular attachment*		
Yes	21	32
No	45	68
*Vertical attachment configuration*		
3 mm	8	38.1
4 mm	9	42.9
5 mm	4	19.0
*Number of aligners*		
Mean (range)	27.6 (14–58)	
*ClinCheck*^*®*^* predicted root tip*		
Mean (range)	7.8° (5–17.3°)	

### Study procedure

Pre-treatment (T0) and predicted post-treatment (T1) digital models of the initial and final stages of each patients’ treatment plan were obtained for each patient and exported as stereolithography (STL) files through the proprietary software for Invisalign^®^ ClinCheck^®^ (Align Technology, San Jose, California, USA). A post-treatment (T2) “achieved” intraoral scan, showing the actual occlusal outcome, was also obtained at the end of the initial series of aligners. The T0 and T1 models were registered in 3D (*i.e.* superimposed) to analyse the predicted change in lower incisor tip. Likewise, the T0 and T2 models were registered to analyse the achieved change in lower incisor tip.

### Individual Cartesian coordinate system

**Fig. 1:**
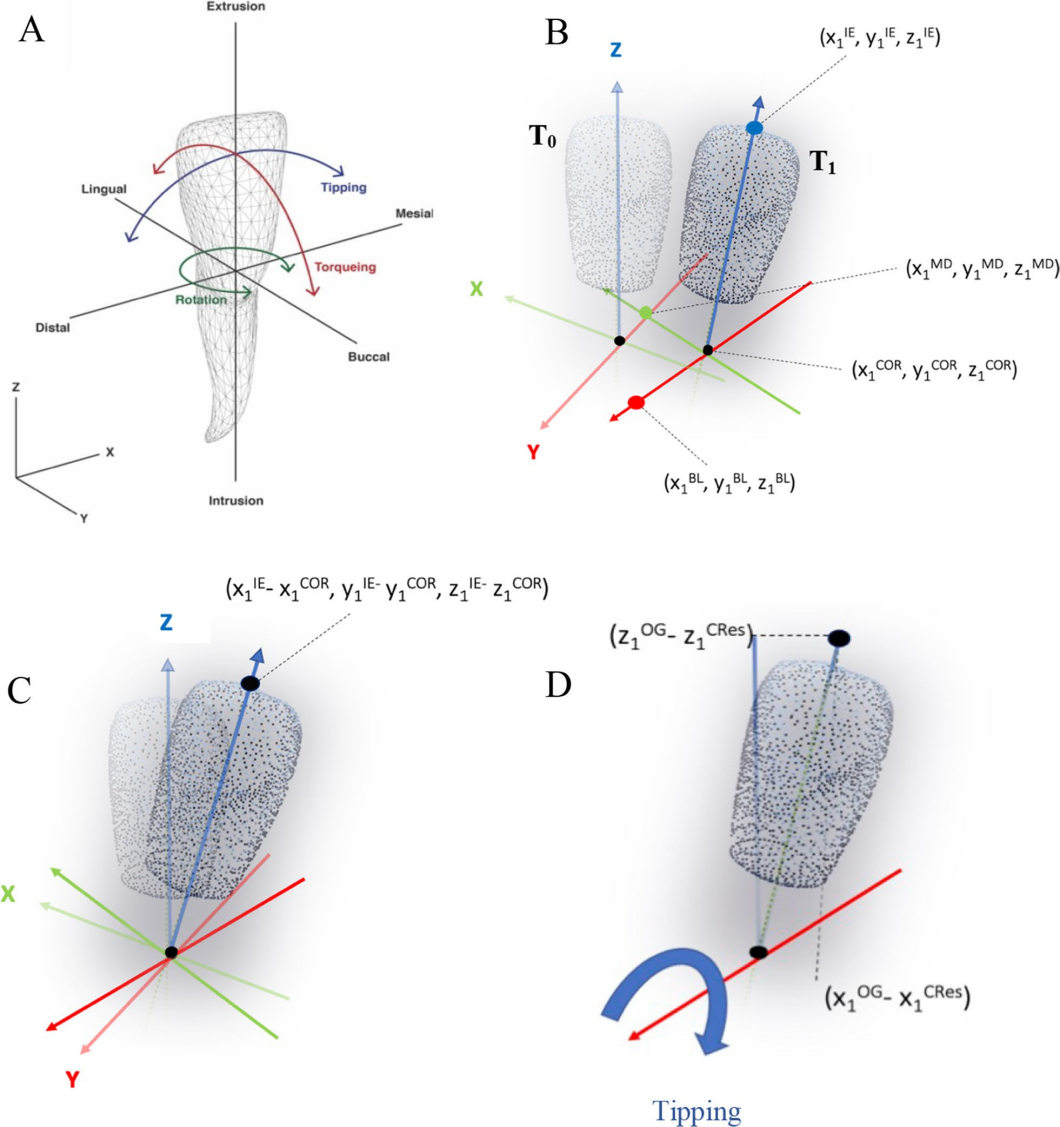
Method used to measure lower incisor tip: **A** diagrammatic drawing showing tooth movement as described with six degrees of freedom. The axes are orientated so that the *x*-axis represented mesio-distal (MD), the y-axis represented bucco-lingual (BL), and the *z*-axis represented occluso-gingival (OG) directions. **B** The C_Res_ was placed at a set distance from the intersection of the crown mesh and the long axis of the tooth (black dot). Arbitrary points were also placed at set distances along the *x*, *y* and* z *axes. **C** The origin of the three axes at T1 reorientated to coincide with C_Res_ of T0. **D** Tipping movement assessed as the rotation around the *y* (bucco-lingual)-axis was then calculated using trigonometry

To quantitatively describe tooth movement, individual teeth were aligned to a Cartesian coordinate system using Geomagic^®^ Control X metrology software (Geomagic, Morrisville, NC, USA). A single operator (JS) placed a reference coordinate system with the origin (0, 0, 0) at the estimated centre of resistance (C_Res_) of the T0 lower incisor. The C_Res_ was traced at approximately two-thirds of the estimated root length (21 mm) along the long axis of the tooth. The long axis was calculated by interpolating multiple cross-sectional areas of the tooth crown surface mesh. This function is built into the software, and thus the tooth mesh was selected in its entirety using the flood selection tool and a vector extracted to represent the long axis. The axes of the coordinate system were orientated so that the *x*-axis represented mesio-distal, the *y*-axis represented bucco-lingual, and the *z*-axis represented occluso-gingival directions for each tooth. The axes were set by the operator using reference planes. Once the Cartesian coordinate system was set, the model was aligned to this coordinate system so that any future models registered to this model would be located within the same coordinate system. Only one origin could be set for alignment; thus, if multiple incisors were eligible, the above process and below registration process were repeated for each incisor. Reference points were placed at set distances along the three axes to describe the tooth movement with six degrees of freedom (Fig. 1).

### Digital model registration

The predicted post-treatment and achieved post-treatment models were individually registered on the pre-treatment model using Geomagic Control X^®^ metrology software using the best-fit surface registration (global and fine) feature with a 50-iteration count. The registration was further refined by using best-fit on the posterior molar occlusal surfaces with minimal movement planned.

### Outcome variables

The change in position from T0 to T1 (predicted) and T0 to T2 (achieved) was measured from C_Res_ and described with six degrees of freedom. Incisor tip was calculated as rotation about the *y* (bucco-lingual)-axes (Fig. 1). This was calculated by subtracting the translational movement of the C_Res_ and then using trigonometry to calculate the rotation, using the following equation:

$$R_{y}^\circ = \tan^{ - 1} \left( {{{x_{1}^{{{\text{OG}}}} - x_{1}^{{{\text{CRes}}}} } \mathord{\left/ {\vphantom {{x_{1}^{{{\text{OG}}}} - x_{1}^{{{\text{CRes}}}} } {z_{1}^{{{\text{OG}}}} - z_{1}^{{{\text{CRes}}}} }}} \right. \kern-\nulldelimiterspace} {z_{1}^{{{\text{OG}}}} - z_{1}^{{{\text{CRes}}}} }}} \right)$$

To describe the type of OTM, a reference point was placed at the incisal edge (IE) by intersecting the long axis and the surface mesh of the crown. A second point was placed 21 mm from the incisal edge along the long axis of the tooth, representing the estimated apex of the tooth (AP). The ratio of AP/IE translation along the frontal plane (*x*-axis) was then used to plot the C_Rot_ relative to the estimated C_Res_ and could be used to classify OTM type as either translation (TR), root movement (RM), controlled tipping (CT), uncontrolled tipping (UCT) or clinically insignificant movement (CIM). The classification criteria are shown in Table 2. The relationship between C_Rot_ and C_Res_ is commonly used to descriptively define different types of tooth movement [22, 29]. The transition from one type of tooth movement to another is, however, currently undefined. For example, CT is defined as when the C_Rot_ is at the apex of the tooth [22, 29]. However, when the C_Rot_ lies somewhere between the apex of the tooth, CT, and the negative infinity, TR, the type of tooth movement or transition point for defining the tooth movement type is unknown. For this reason, arbitrary thresholds were defined to describe transition between different types of tooth movements. For the analysis and discussion, RM and TR are grouped together as both movements require a high moment/force (M/F) ratio which is accompanied by pronounced movement of the root.

**Table 2:** Classification of OTM type

	Classification of OTM	Explanation	AP/IE ratio	Distance of *C*_Rot_ from *C*_Res_
	Root movement	*C*_Rot_ close to incisal edge, displacement of IE is minimal	IF AP/IE < − 1.5 OR AP/IE > 2.4 AND “Ap movement” > 0.5 mm	6 < *C*_Rot_ < 30
	Uncontrolled tipping	*C*_Rot_ close to *C*_Res_, displacement of IE and AP similar in magnitude but opposite in direction	IF − 1.5 < AP/IE < −0.2 AND “Ap movement” > 0.5 mm AND “IE movement” > 0.5 mm	−3 < *C*_Rot_ < 6
	Controlled tipping	*C*_Rot_ close to apex, displacement of Ap is minimal	IF − 0.2 < AP/IE < 0.5 AND “IE movement” > 0.5 mm	30 < *C*_Rot_ < −3
	Translation	*C*_Rot_ approaching infinity, displacement of Ap and IE similar in magnitude and direction	IF 0.5 < AP/IE < 2.4 AND “Ap movement” > 0.5 mm AND “IE movement” > 0.5 mm	*C*_Rot_ > 30 OR *C*_Rot_ < −30
	Clinically insignificant movement (CIM)	A/IE fits within above category but Apex AND/OR IE movement < 0.5 mm		

Arbitrary thresholds based on distances between *C*_Rot_ and *C*_Res_ were used to describe transition between different types of tooth movements

The percentage of accuracy was calculated as:

$${\text{Percentage}}\;{\text{of}}\;{\text{accuracy}} = 100\% - \left( {\left[ {{{\left( {\text{predicted - achieved}} \right)} \mathord{\left/ {\vphantom {{\left( {\text{predicted - achieved}} \right)} {{\text{predicted}}}}} \right. \kern-\nulldelimiterspace} {{\text{predicted}}}}} \right] \times 100\% } \right)$$

Directionality was accounted for; however, no allowance was made for percentages above or below 100%. This allowed for incisors that have considerably under tracked or even over tracked the predicted movements to be included.

### Error study

To determine intra-examiner reliability, 25% of the sample was randomly selected using the random number generator in Microsoft Excel software (version 16.52). After a washout period of two weeks, 17 incisors were remeasured. The error of the method for each variable (root tip predicted and root tip achieved) was calculated using the intraclass correlation coefficient and the Dahlberg formula [30].

### Statistical analysis

Descriptive statistics was used to estimate means, ranges and standard deviations for each outcome measured. Absolute values were used for mean calculations as the predicted and achieved values included both positive and negative values indicating direction. This prevents the mean averaging close to zero and misleadingly or inflating the accuracy level.

Relationships between predicted and achieved changes for angular and linear measurements were described using scatter plots, linear regression analyses and Bland–Altman plots. Cohen’s kappa was used to estimate the agreement between the predicted and achieved movement types [31].

Pearson’s correlation coefficients and coefficients of determinations (*R*^2^) were used to test the correlation between different variables and the amount of variance attributed to the relationship. Linear mixed model analyses were used to test the influence of rectangular attachments on root tip, apex movement and consequently movement type after controlling for wear time and the number of aligners. To account for a small number of repeated measurements from multiple teeth obtained from the same patient, a random term was entered to identify patients.

Statistical analyses were performed using SPSS software (version 20.0, IBM Corporation, Chicago, Illinois, USA). The level of statistical significance was set at *p* ≤ 0.05.

## Results

The intraclass correlation coefficients (ICCs) for predicted and achieved root tip varied between 0.97 and 0.99, demonstrating excellent intra-examiner reliability. Likewise, the Dahlberg error ranged from 0.1 to 0.6 degrees for predicted and achieved root tip demonstrating excellent reproducibility.

**Fig. 2:**
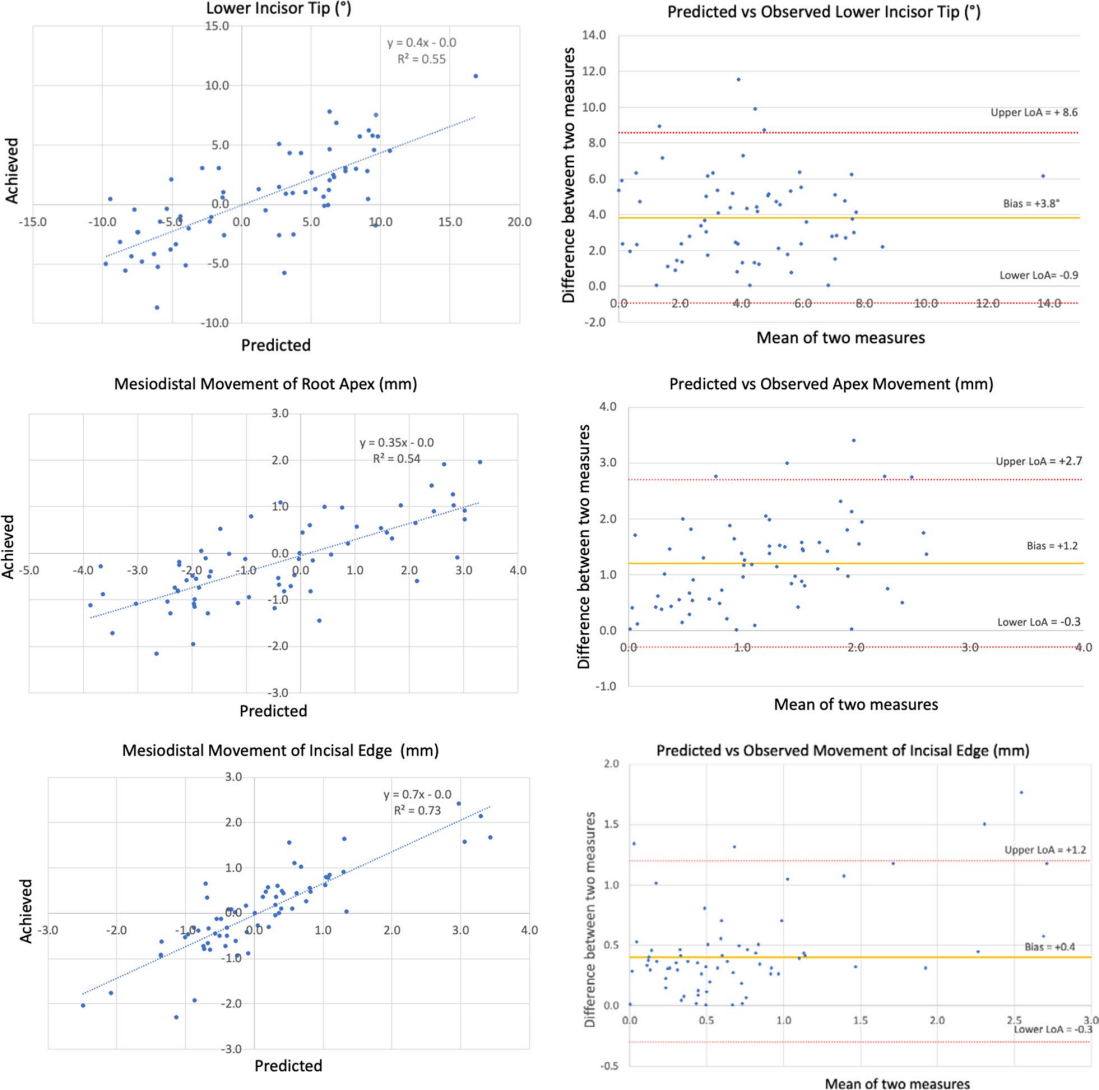
Scatterplots of predicted values versus achieved values for lower incisor tip, mesio-distal movement of root apex and incisal edge movements. Scatterplot data (charts to the left) were fitted using linear regression analysis and the regression coefficients are given in the charts, including the coefficient of determinations (*R*^2^). The best linear fitting was obtained from the mesio-distal movement of incisal edge (*R*^2^ = 0.73), while the association between predicted and observed values was weaker for the lower incisor tip and the movement of root apex with *R*^2^ values of 0.55 and 0.54, respectively. Bland–Altman plots were used to describe systematic differences between predicted and observed measurements. The difference between each pair of predicted and observed values was plotted against their mean value. This allowed estimate the mean bias between predicted and observed values and their upper and lower 95% limits of agreement (LoA)

The relationship between predicted and achieved movements is described using scatter diagrams, linear regression equations and Bland–Altman plots (Fig. 2). A significant positive correlation was found between predicted incisor tip and achieved incisor tip (*R*^2^ = 0.55; *p* < 0.001). The regression coefficient shows that for every degree of tip predicted, 0.4 degrees of tip was achieved. The strongest correlation was found for predicted and achieved movement of the incisal edge (*R*^2^ = 0.73; *p* < 0.001). For every 1 mm of incisal edge movement planned, 0.7 mm was clinically achieved. A weaker correlation (*R*^2^ = 0.54; *p* < 0.001) was found for movement of the apex. For every 1 mm of apex movement planned, 0.4 mm was achieved.

The mean difference between predicted and achieved incisor tip was 2.8 degrees (SD 2.7). The mixed model analysis showed that incisor tip, as represented by the absolute value, was not influenced by the number of aligners worn (*F* = 0.5; *p* = 0.499) or the wear time (*F* = 0.7; *p* = 0.424). On average, 1.2 degrees greater mean incisor tip was achieved in the attachment group than no attachment group; however, this was not statistically significant (*F* = 3.7; *p* = 0.062).

The movement of the apex (absolute value) was also not influenced by the number of aligners worn (*F* = 1.0; *p* = 0.313) or the wear time (*F* = 0.1; *p* = 0.763), but it was significantly influenced by the vertical attachments (*F* = 4.3; *p* = 0.042). The presence of a vertical attachment resulted in 0.5 mm (95% confidence intervals 0.2–0.9 mm) greater apex movement than the no attachment group.

The accuracy of predicted movement showed a large variation from -187% to 217%. The negative percentage of accuracy reflects 12 incisors (18%), which moved in the opposite direction to the planned movement. The type of OTM achieved was also likely different to that predicted (Kappa = 0.13, 95% CI 0.01–0.26; chi-square *p* = 0.054). Table 3 presents a cross-tabulation of the results. Forty-five incisors (67%) were predicted to move by RM/TR, while 30 incisors (46%) achieved this type of OTM. When incisors were programmed for CT, 17% followed this type of tooth movement, while 25% showed UCT. Seventeen incisors (26%) were classified as achieving CIM as there was insufficient movement of the crown or apex to meet the classification criteria.

**Table 3:** Cross-tabulation of predicted and achieved movement types

		Achieved				
		CIM	CT	RM/TR	UCT	Total
Predicted	CIM	0	0	2	1	3
	CT	4	2	3	3	12
	RM/TR	12	2	25	6	45
	UCT	1	3	0	2	6
	Total	17	7	30	12	66

Movement classified as: clinically insignificant movement (CIM), controlled tipping (CT), root movement (RM)/translation (TR) and uncontrolled tipping (UCT). Each cell count represents the number of incisors classified for each movement type. All types of OTM predicted were included in the analysis as in all cases the ClinCheck^®^ reported greater than 5 degrees of root tip

## Discussion

Lower incisors frequently present with incorrect root positioning and require root tip as part of comprehensive orthodontic treatment. Adequate uprighting of lower incisors is particularly critical for gaining space (as a mesio-distally tipped incisor occupies more space than an upright incisor) and reducing the appearance of open gingival embrasures [2]. The present study aimed to determine the predictability of lower incisor tip with the Invisalign^®^ appliance and to quantify the influence of vertical attachments on the expression of incisor tip. All incisors included in the study had at least five degrees of root tip programmed in the ClinCheck^®^ software TMT. The majority of incisors achieved some form of controlled root movement (56% either classified as CT or RM/TR); however, the type of OTM achieved was unpredictable. The efficacy of root movement was significantly less than planned, although the presence of an attachment did improve the mesio-distal translation of the simulated root apex.

### Predictability of lower incisor root tip

A threshold of five degrees of lower incisor root tip was chosen for this study as previous studies have demonstrated an average length of a lower incisor to be 21 mm [32], which would equate to 1.8 mm of apex movement. This was considered clinically relevant, although this is subjective. There was only a weak correlation between the ClinCheck^®^ software predicted root tip value from the TMT and our methodology used to calculate incisor tip (*R*^2^ = 0.30). As a result, the type of OTM of three incisors included in the study was classified as “clinically insignificant movement” for the predicted movement type despite the TMT reporting at least five degrees of root tip. It is currently unknown how prescribed root movements are calculated in the TMT, and clinicians should interpret this table with some caution.

Mesio-distal translation of the incisal edge showed the highest predictability, with 0.7 mm achieved for every 1 mm of movement predicted. The region of the aligner covering the incisal edge is the thickest and subsequentially the strongest area and thus can engage and control this part of the tooth with more accuracy. Using the incisal edge as a reference point to measure tooth movement can led to an inflated assumption of tooth movement. This has been demonstrated with previous studies that show better predictability of the cusp tip/incisal edge compared to the centroid or root of the tooth [17, 18]. The accuracy of lower incisor crown tip with Invisalign^®^ has been reported in the range of 38.5–51.5% [13]. These studies have looked at crown tip only, and hence, there can be no direct comparison to the movements measured from the C_Res_, presented in this study.

The overall accuracy of lower incisor tip in this study was 35%. Nonetheless, this mean value conceals substantial individual variation that was observed (-187 to 217%). The negative values of accuracy represent cases where the achieved tip occurred in the opposite direction to what was planned. This occurred in a relatively large proportion of the sample (18%). A possible explanation for this was demonstrated in one case where a type C anchorage was planned with the lower posterior segments mesialising. A loss of anterior anchorage appeared to have caused distal crown tipping of the lower incisors rather than the planned mesial tip. A study looking at Invisalign^®^ G6 setup for first premolar extractions showed a similar loss of anchorage with first molars that were planned to tip distally instead tipping mesially and translating more than predicted [33].

The results of this study did not indicate a significant difference between 1-weekly or 2-weekly wear protocols and the expression of lower incisor root tip. The majority of cases (71%) included were 2-weekly aligner changes; hence, there may have been insufficient power to detect a difference. Furthermore, some clinicians change aligners weekly but halve the tooth movement velocity, and thus, the rate of tooth movement is the same as 2-weekly change protocol making interpretation of this challenging.

### Describing orthodontic tooth movement

Improving our understanding of OTM with aligners is critical in order to move teeth more predictably and efficiently. A descriptive analysis of tooth movement types was attempted using the ratio of movement of the incisal edge reference point (IE) and the apex reference point (AP). This method enabled the C_Rot_ to be plotted relative to the estimated C_Res_ and the type of tooth movement defined. There is often inconsistency in the literature regarding the type of tooth movement achieved with CAT. Regarding root movement, torque has previously been reported as a change in angulation in the sagittal dimension when in actual fact the same change in angulation could have been achieved through uncontrolled tipping [28, 34]. In this study, the equivalent of torque in the frontal plane, root tip, was defined as when the C_Rot_ was close to the incisal edge and the displacement of IE was minimal. The AP movement needed to be equal or greater than 0.5 mm to satisfy this movement type.

Our results demonstrated a poor correlation between predicted and achieved movement types. This could be attributed to the large number of achieved cases being classified as CIM (26%), as IE or AP did not achieve 0.5 mm of movement despite the ratio reflecting an alternative OTM type. Once again, this highlights the under-expression of tooth movement clinically achieved compared to digitally prescribed.

The lack of correlation between predicted and achieved movement types was also reported by previous researchers [16], who looked at pre- and post-treatment cone beam computer tomography (CBCT) to determine the type of OTM occurring in the sagittal plane. In contrast, they found when root torque was programmed 100% of teeth achieved this type of tooth movement. In this study, when root movement was programmed, only 56% of incisors achieved this type of movement.

In our study significantly more of the achieved movements were classified as uncontrolled tipping (18%), compared to predicted movements (9%). Similarly, a second study used CBCT images to assess the type of OTM occurring with CAT over an 8-week period during which bodily protraction of a central incisor was planned [18]. In this study, CBCT imaging illustrated the crown and root moving in opposite directions, exhibiting OTM by uncontrolled tipping [18].

It was previously thought that CAT could be more effective than fixed appliances at root movement as the crown is encased in the aligner, and thus, the distance between two potential points of force application could be further increasing the moment arm. The limitation of this theory is the ability of aligners to generate adequate force levels near the cervical margin. Experimental models have shown the applied force at the free gingival margin of an aligner to be insufficient to generate a counter moment. Thus, the force application differential ultimately favours a tipping moment [35, 36].

When the type of tooth movement is considered, the efficacy of tooth movement showed an interesting finding. Of the cases where RM was programmed and achieved, the accuracy was 37.9%. In comparison, for CT, the accuracy was 49.7%, and for UCT, 74.7%. This finding suggests that when the type of OTM predicted is achieved, the type of OTM may determine the amount or accuracy of the predicted tooth movement that is clinically achieved, with more difficult movements achieving less accuracy. Given the complex biological interactions that are required for OTM (particularly root movement which requires more bone resorption and deposition), it is virtually inevitable that some inaccuracies will be present. Just like the torque over-correction already built into pre-adjusted edgewise appliances, clinicians should be prepared for shortfall when programming root movements with CAT.

### Influence of vertical rectangular attachments

Composite attachments bonded to the tooth surface increase the surface area available for an aligner to engage and exert force on a tooth [37]. They also theoretically make more complex tooth movements possible with CAT [38–41]. In our study, the presence of a vertically orientated rectangular attachment resulted in significantly more translation of the experimental root apex (0.5 mm; CI 0.2–0.9 mm). An additional 1.2 degrees of root tip was achieved with an attachment; however, this was not statistically significant. The greater apex translation is small but clinically relevant and provide clinicians with an initial recommendation for adding vertical attachments to lower incisors requiring considerable root movement.

The presence of an attachment, however, had no influence on the type of OTM achieved as represented by absolute tip. This was an unexpected finding, as previous studies having reported attachments are required to generate a force couple to prevent simple tipping movements [25, 42]. It is feasible that individual crown morphology and the accessibility of the mesial and distal contact surfaces for the aligner to push against (*i.e.* due to a labially positioned incisor or a staging pattern that first creates interproximal spaces) may also be sufficient to generate a counter moment and improve the predictability of OTM with aligners. The width and height of each individual clinical crown will also determine the amount of surface contact of the aligner to the tooth crown and hence will influence OTM. In addition, Invisalign^®^ rectangular attachments are available in 3, 4 and 5 mm configurations. Increasing the length of the attachment would potentially increase the moment arm of the couple, thus generating a more effective moment for root movement. Due to the limited sample size, no differentiation of attachment size could be made in the analysis. As only one-third of the sample had an attachment placed, it is also possible that there is insufficient power to detect a difference in the type of OTM. Furthermore, no allowance could be made for attachments that may have worn or completely debonded during the treatment.

Previous studies reporting accuracy of tooth movements have often relied on an experienced orthodontist to make decisions regarding the choice of optimised and/or conventional attachments, with little details provided [8, 14]. In order for CAT to be truly customised to the patient and optimised for the type of OTM required, further reporting on attachments should be attempted.

### Limitations

The present study has several limitations. Firstly, the data were retrospectively collected from non-extraction adult patients, thus limiting the external validity of the results. Adult patients were selected for this study to reduce the effect of growth and the difficulties that arise with superimpositions of dentitions in growing patients. Adult patients also represent the majority of Invisalign^®^ treatments; therefore, despite applying strict inclusion/exclusion criteria an adequately large sample size could still be collected. As only Invisalign^®^ aligners were include in this study, the results cannot be generalised to other aligner systems. Furthermore, as only non-extraction cases were included, the results cannot be applied to extraction cases where incisor root tip control may be more challenging.

The superimposition technique chosen was based on a previously reported protocol by Grünheid et al. [43] using best-fit (global and fine) registration. This was further refined by including cases with only minimal sagittal movement of mandibular molars. Unlike the palatal rugae in the maxilla, no stable structures have been reported for superimposition of the mandible. Furthermore, when using predicted models from the ClinCheck^®^ software, only clinical crowns are available for superimposition. In the future, anatomical landmarks on CBCTs may improve the accuracy of superimpositions.

The vector representing the long axis of the tooth may not represent the true long axis of the crown and root. However, as this vector is based on the surface mesh of the crown, which is not altered by treatment, the vector is reproducible and can be used to simulate the change in root movement. Significant amounts of anterior IPR, anterior restorations during treatment or attachments remaining in the final scan were therefore excluded to avoid any potential changes in the crown mesh. This unique methodology improves our understanding of how teeth move with aligners. Moreover, by measuring OTM from the estimated C_Res_ rather than the incisal edge, tooth displacement is not influenced by simple tipping movements [10]. It was assumed that the C_Res_ was 14.5 mm from the incisal edge as experimentally C_Res_ has been estimated at approximately two-thirds of the root length for incisors [44]. However, accurate placement of C_Res_ was not an objective of this study since individual factors such as periodontal support, attachment levels and root morphology will influence this position [44, 45]. Given the numerous assumptions made regarding the position of C_Res_, the exact magnitude and type of OTM should be interpreted with caution. These same individual factors as well as bone biology and incisor crown anatomy could also influence that accuracy of CAT to express the desired tooth movements. Such factors could not be controlled for in this retrospective study.

Previous studies have used CBCTs to access root movement more accurately [18], though the sample sizes are often small due to the increased radiation exposure. For the patients included in this study, the diagnosis and treatment planning would not have been altered by taking CBCTs pre-treatment and could not be justified post-treatment while adhering to ALARA (as low as reasonably achievable) principles. In future, graphic modelling of root positioning from crown data may provide a means to predict root movements more reliably without further radiation exposure to the patient [46].

Finally, as with almost every study looking at the predictability of tooth movements using clear aligners, multiple teeth from the same patient were included. This presents a limitation as one tooth cannot reasonably move without an effect on adjacent teeth or anchorage units. This study has limited this as much as possible by only looking at one type of tooth movement: mesio-distal tip on lower incisors. However, some patients had multiple incisors included.

## Conclusion

The primary objective of this study was to quantify how much of the predicted lower incisor tip is achieved clinically and attempt to describe the type of OTM achieved with aligners. Given the limitations of the present study, it is difficult to incorporate a clinical recommendation in the conclusion, though our findings may support a need to plan for over-correction at the end stages of treatment. Interestingly, using the classification of OTM type, most incisors did achieve root movement. Thus, within the limitations of the study, it is possible to move roots using Invisalign^®^ but not as predictably as ClinCheck^®^ suggests. Moreover, the amount of root movement achieved was on average substantially less than predicted. Vertical rectangular attachments are recommended when large amounts of root movement are planned, and their presence improves the ability to translate the root apex. Future research should be directed at lower incisor extraction cases in which large amounts of root movement are required to achieve root parallelism and stable treatment outcomes. A better understanding of OTM may be obtained if sequential digital scans are taken throughout the treatment rather than from only two time points. This would enable further assessment of the pattern of OTM with aligners.

## Data Availability

The datasets collected and analysed during the current study are available from the corresponding author on reasonable request.

## Abbreviations

3D: Three-dimensional
AP: Apex
BL: Bucco-lingual
CAT: Clear aligner treatment
CBCT: Cone beam computed tomography
CIM: Clinically insignificant movement
C_Res_: Centre of resistance
C_Rot_: Centre of rotation
CT: Controlled tipping
IE: Incisal edge
IPR: Interproximal reduction
MD: Mesio-distal
M/F: Moment-to-force ratio
OG: Occluso-gingival
OTM: Orthodontic tooth movement
RM: Root movement
STL: Stereolithography (file)
T0: Pre-treatment
T1: Predicted post-treatment
T2: Achieved post-treatment
TR: Translation
TMT: Tooth movements table
UCT: Uncontrolled tipping
